# White Matter Fractional Anisotropy Is a Superior Predictor for Cognitive Impairment Than Brain Volumes in Older Adults With Confluent White Matter Hyperintensities

**DOI:** 10.3389/fpsyt.2021.633811

**Published:** 2021-05-05

**Authors:** Yi Xing, Jianwei Yang, Aihong Zhou, Fen Wang, Cuibai Wei, Yi Tang, Jianping Jia

**Affiliations:** ^1^Department of Neurology, Innovation Center for Neurological Disorders, National Clinical Research Center for Geriatric Disorders, Xuanwu Hospital, Capital Medical University, Beijing, China; ^2^Key Laboratory of Neurodegenerative Diseases, Ministry of Education of the People's Republic of China, Beijing, China

**Keywords:** white matter hyperintensities, vascular cognitive impairment, fractional anisotropy, brain volume, hippocampus, nucleus basalis of Meynert

## Abstract

Older patients with confluent white matter hyperintensities (WMHs) on magnetic resonance imaging have an increased risk for the onset of vascular cognitive impairment (VCI). This study investigates the predictive effects of the white matter (WM) fractional anisotropy (FA) and brain volumes on cognitive impairment for those with confluent WMHs. This study enrolled 77 participants with confluent WMHs (Fazekas grade 2 or 3), including 44 with VCI-no dementia (VCIND) and 33 with normal cognition (NC). The mean FA of 20 WM tracts was calculated to evaluate the global WM microstructural integrity, and major WM tracts were reconstructed using probabilistic tractography. Voxel-based morphometry was used to calculate brain volumes for the total gray matter (GM), the hippocampus, and the nucleus basalis of Meynert (NbM). All volumetric assays were corrected for total intracranial volume. All regression analyses were adjusted for age, gender, education, and apolipoprotein E (ApoE) gene ε4 status. Logistic regression analysis revealed that the mean FA value for global WM was the only independent risk factor for VCI (z score of FA: OR = 4.649, 95%CI 1.576–13.712, *p* = 0.005). The tract-specific FAs were not associated with the risk of cognitive impairment after controlling the mean FA for global WM. The mean FA value was significantly associated with scores of Mini-Mental State Examination (MMSE) and Auditory Verbal Learning Test. A lower FA was also associated with smaller volumes of total GM, hippocampus, and NbM. However, brain volumes were not found to be directly related to cognitive performances, except for an association between the hippocampal volume and MMSE. In conclusion, the mean FA for global WM microstructural integrity is a superior predictor for cognitive impairment than tract-specific FA and brain volumes in people with confluent WMHs.

## Introduction

Vascular cognitive impairment (VCI) is the second most common cause of acquired cognitive impairment, behind only Alzheimer's disease (AD), with subcortical ischemic VCI (SIVCI) caused by cerebral small vessel disease (SVD) as its most common subtype (1). White matter lesions (WMLs), which present as white matter hyperintensities (WMHs) on magnetic resonance imaging (MRI), are one of the primary manifestations of SVD. The appearance of WMHs varies from small punctures, to diffuse and confluent lesions. It has been demonstrated that WMHs and VCI are associated in a severity-dependent manner. Compared to patients with mild WMHs, those with severe WMHs experienced a three-fold increase in the risk of vascular dementia in a 3-year follow-up study (2). This association sparked the suggestion that preventive trials for VCI should focus on patients with confluent WMHs (3). However, although older adults with confluent WMHs are more likely to have VCI, the risk is disparate among individuals. Within the same severity of WMHs, some patients rapidly develop cognitive impairment while others maintain normal cognition for an extensive period. Identifying the predictors of cognitive impairment, especially in those who already have confluent WMHs, would provide a crucial tool for early detection and prevention.

Many previous studies have shown that the MRI metrics for WMHs are useful predictors for cognitive decline in the elderly, with the severity of WMHs, including WMHs visual rating (2, 4) and the volume of WMHs (5, 6), serving as the defining attributes. However, among patients who already possess confluent WMHs, these MRI metrics do not appear sensitive enough to identify those at a higher risk of future cognitive impairment (7, 8). Diffusion tensor imaging (DTI), which measures the restricted diffusion of water in tissue, is a more sensitive method to evaluate WMLs. DTI has been used to estimate WM microstructural integrity and find abnormalities even when the WM appeared normal *via* routine MRI (9). For patients with SVD, studies using DTI have found alternations in the WM microstructure spanning almost the entire cerebral WM (10). One longitudinal study for SVD patients who presented lacunar infarcts and confluent WMHs on MRI, demonstrated that WM microstructural changes on DTI were correlated with cognitive decline (11). Thus, if DTI can discern the differences between WM microstructures which are linked to cognitive decline, then it has exciting potential as a predictor for cognitive impairment in people with confluent WMHs.

In addition to WMHs, gray matter (GM) and hippocampal atrophy are also used as common predictors for cognitive impairment in the elderly. More specifically, some studies on people with WMHs have shown that the severity/volume of WMHs and volumes of the total GM and the hippocampus are independent predictors for cognitive decline (2, 5, 6). However, these results are not universal and other studies have indicated that WMHs volume is not associated with cognitive performances, once GM atrophy is accounted for in regression models (7, 8). The inconsistency in results is likely due to methodological heterogeneity, such as a wide spectrum of WMHs and different MRI indices. Thus, for those with confluent WMHs it remains uncertain which is the strongest MRI predictor of VCI among DTI metrics and/or GM/hippocampal volumes. Additionally, the nucleus basalis of Meynert (NbM) has been discussed as another potential marker, as it is another important cognitive brain region and previous studies have shown that NbM atrophy is a sensitive marker for cognitive impairment in AD and Parkinson's disease (12, 13). Cholinergic neurons play key roles in cognitive function, and the NbM is the largest cholinergic nucleus in the basal forebrain. It is unclear if the volume of NbM could be used as a predictor of cognitive impairment in people with confluent WMHs.

This study aims to identify the predictors of cognitive impairment in older adults with confluent WMHs. The candidate predictors are DTI metrics and the volumes of various brain regions including the total GM, hippocampus and NbM. Since previous studies have demonstrated that there are associations between WMHs and GM atrophy (14, 15), our study will also analyze the associations between WM integrity and brain volumes.

## Materials and Methods

### Participants

We consecutively enrolled participants with confluent WMHs and normal cognition (NC) or VCI-no dementia (VCIND) from the Department of Neurology at Xuanwu Hospital. The inclusion criteria included: (1) age ≥55, and ≤ 75 years; (2) literate in Han Chinese and right-handed; (3) confluent WMHs viewed on MRI defined as Fazekas grade 2 or 3 (16), without cortical or watershed infarcts, hemorrhages, or hydrocephalus; and (4) no or very mild hippocampal or entorhinal cortex atrophy (the medial temporal lobe atrophy scale: 0 or 1) (17). The participants without cognitive impairment complaints and a clinical dementia rating (CDR = 0) were classified as NC.

The diagnosis criteria of VCIND were the same as our previous study (18). Briefly, all patients were determined by a consensus panel including three senior neurologists and met the following inclusion criteria: (1) Complaint/informant report of cognitive impairment involving memory and/or other cognitive domains with a duration of at least 3 months. (2) The patients were neither normal nor demented, as defined by the Diagnostic and Statistical Manual of Mental Disorders, Fourth Edition [a CDR of ≥0.5 on at least one domain, a global score of ≤ 0.5 (19), and a Mini-Mental State Examination (MMSE) score of ≥20 (primary school) or ≥24 (junior school or above) (20, 21)]. (3) All patients had normal or slightly impaired daily living activities, as defined by a total score of ≤ 1.5 for the three functional CDR domains (home and hobbies, community affairs, and personal care).

Exclusion criteria included: (1) WMLs with specific causes (e.g., multiple sclerosis); (2) severe aphasia, physical disabilities, or any other factor that may preclude completion of neuropsychological testing; (3) medical history of stroke with focal neurological features and subsequent MRI lesions; (4) other disorders or use of medication that might affect cognitive functions; (5) clinically significant gastrointestinal, renal, hepatic, respiratory, infectious, endocrine, or cardiovascular diseases as well as cancer, alcoholism, or drug addiction; and (6) psychiatric disorders.

Written informed consent was obtained from all participants. This study was approved by the Institutional Review Board of Xuanwu Hospital.

### Assessments

The MMSE was used to assess global cognitive ability. The World Health Organization–University of California–Los Angeles Auditory Verbal Learning Test (WHO-UCLA AVLT) was used to measure memory function. This test included immediate recall (maximum score = 45), long delayed free recall (maximum score = 15) and long delayed recognition (maximum score = 15), in which higher values represent a better performance (22). The Trail-Making Test (TMT) B minus A (B-A) score was applied to evaluate executive function. Language function was measured by the Boston Naming Test (BNT).

The apolipoprotein E (ApoE) genotypes were tested by the restriction enzyme digestion approach as has been previously described (23). All participants were defined as ApoE ε4 positive if they carried at least one copy of the four alleles.

### MRI Data Collection

The MRI data were acquired using a 3.0 T Siemens scanner. High-resolution T1-weighted images of the whole brain were obtained using a sagittal 3D magnetization prepared rapid gradient echo (MP-RAGE) sequence: repetition time (TR) 1,690 ms, echo time (TE) 2.56 ms, slice thickness 1 mm, flip angle 12°, field of view (FOV) 256 × 256 mm^2^. Matrix = 256 × 256, slice number = 176.

3D Flair images were obtained with the following settings: TR 5,000 ms, TE 392 ms, inversion time 1,800 ms, FOV 240 × 240 mm^2^, slice thickness 1 mm, and flip angle 120°.

DTI images were acquired using a diffusion-weighted double spin-echo EPI sequence: TR 8,000 ms, TE 96 ms, 64 diffusion weighted directions with a b value of 1,000 s/mm^2^ and 11 images with a b value of 0 s/mm^2^, flip angle 90°, FOV 224 × 224 mm^2^, in-plane resolution 1.75 × 1.75 mm^2^ voxels, and 54 contiguous 2-mm thick axial slices.

### WM Fractional Anisotropy

Fractional anisotropy (FA), the most widely used metric of DTI, was analyzed in this study to investigate the WM microstructure. DTI data processing was implemented using PANDA software (a pipeline tool for analyzing brain diffusion images; http://www.nitrc.org/projects/panda/) (24). The preprocessing steps were as follows: converting DICOM files into NIfTI images, estimating the brain mask, removing non-brain tissue, correcting eddy current and head motion, adjusting the diffusion gradient direction, and then calculating DTI metrics. After preprocessing, individual FA images of native space were non-linearly registered the FA standard template in the Montreal Neurological Institute (MNI) space. Atlas-based analysis was used, and PANDA calculated the regional FA by averaging the values within each region of the JHU WM Tractography Atlas (25). The regions of interests (ROIs) included anterior thalamic radiation, corticospinal tract, cingulum (cingulate gyrus), cingulum (hippocampus), forceps major, forceps minor, inferior fronto-occipital fasciculus, inferior longitudinal fasciculus, superior longitudinal fasciculus, uncinate fasciculus, and superior longitudinal fasciculus (temporal part). All of the tracts were evaluated in both the left and right hemispheres, except for the forceps major and forceps minor. A mean FA value of these 20 tract-specific ROIs, representing global WM microstructure, was used in subsequent statistical analyses.

Furthermore, to investigate whether tract-specific FA predicts the onset of cognitive impairment, we used probabilistic tractography to reconstruct major tracts with the same method already published (26). The FA of thirteen major tracts were calculated, including anterior thalamic radiation (ATR), acoustic radiation (ACR), cingulate gyrus part of cingulum (CGC), parahippocampal part of cingulum (CGH), corticospinal tract (CST), forceps major (FMA), forceps minor (FMI), inferior fronto-occipital fasciculus (IFO), inferior longitudinal fasciculus (ILF), posterior thalamic radiation (PTR), superior longitudinal fasciculus (SLF), superior thalamic radiation (STR) and uncinate (UNC).

### Voxel-Based Morphometric Analysis

T1 images were analyzed by SPM8 (Welcome Trust Centre for Neuroimaging, London, UK; https://www.fil.ion.ucl.ac.uk/spm/software/spm8/). They were first segmented into the GM, WM and CSF using the unified segmentation module as implemented in the “new segment” option of SPM8. Then, these tissue segmented images were used to generate a template *via* DARTEL. All individual images were warped to this template and then aligned with the MNI space. Finally, normalized images were smoothed using an 8 mm full-width-half-maximum (FWHM) Gaussian kernel. The volumes of the GM, WM, and CSF were summed to provide an estimate of the total intracranial volume (ICV). The bilateral hippocampal regions were determined *via* the definition of the Automated Anatomical Labeling atlas (27) and their volumes averaged. The NbM template that has been previously reported was used to extract its volume (12, 28). The volumes of the GM, hippocampus and NbM were corrected for ICV.

### Statistical Analysis

#### Predictors for Cognitive Performances

The Kolmogorov-Smirnov test was applied to assess the normality of the continuous variables. To compare the demographic variables, neuropsychological scores, and MRI data, independent sample *t*-tests and chi-squared tests (or Fisher's exact tests, where appropriate) for dichotomous variables were used. If the data showed a skewed distribution, a Mann–Whitney *U* test was employed.

Logistic regression analysis was performed to elucidate the association between the candidate MRI predictors and the risk for onset of cognitive impairment. The disease status (VCIND or NC) was the dependent variable and FA and the volumes of the total GM, hippocampus and NbM were the independent variables. Age, gender, education and ApoE ε4 status were all included in the models as covariates. All MRI predictors were standardized by z score to enable comparisons across variables, prior to the regression analysis. Spearman rank correlation was then used to explore the correlations between the MRI predictors and the performance of each cognitive domain. Based on this exploratory correlation analysis, the later multivariate linear regression models were constructed.

#### Associations Between WM FA and Brain Volume

To explore the associations between FA and brain volumes, linear regression models were developed with FA as the independent variable and the volumes of the total GM, the hippocampus and the NbM as the dependent variables. Adjustments were made for age, gender, education, and ApoE ε4 status. The brain volumes (by tertiles of mean FA) were compared using analysis of variance.

All statistical analyses were completed with SPSS software (Version 23.0. Armonk, NY: IBM Corp). Significance was considered as *p* < 0.05.

## Results

A total of 77 participants with confluent WMHs, including 44 with VCIND and 33 with NC, were recruited into this study. The characteristics of these participants are present in Table 1. There were no group differences in age, gender, education, medical history, or WMHs severity distribution between the VCIND and NC groups. The percentage of ApoE ε4 positivity was notably higher in the VCIND group. Compared to the NC group, participants with VCIND had worse performance in all cognitive tests. The results of mean FA for global WM, tract-specific FAs, actual volumes, and the volume ratios correcting for ICV are also shown in Table 1. The VCIND group had lower mean FA for global WM and FA for ATR, CGC, CST, FMA, FMI, IFO, ILF, PTR, SLF and UNC and smaller volume ratios of total GM, hippocampus and NbM (correcting for ICV) than those in the NC group.

**Table 1:** The characteristic, neuropsychological assessments and MRI results of the participants.

	**Total, *n* = 77**	**VCIND, *n* = 44**	**NC, *n* = 33**	***P***
Age	66.44 ± 6.92	66.05 ± 7.18	66.97 ± 6.62	0.565
Female (*n*, %)	37 (48.1%)	19 (43.2%)	18 (54.5%)	0.323
Education (year)	11.12 ± 2.66	10.64 ± 2.27	11.76 ± 3.02	0.067
Fazekas = 2 (*n*, %)	28 (36.4%)	14 (31.8%)	14 (42.4%)	0.338
**Medical history (*****n*****, %)**				
Hypertension	57 (74.0%)	35 (79.5%)	22 (66.7%)	0.202
Diabetes	19 (24.7%)	10 (22.7%)	9 (27.3%)	0.647
Hyperlipidemia	42 (54.5%)	25 (56.8%)	17 (51.5%)	0.644
ApoE ε4 positive (*n*, %)	12 (15.6%)	10 (22.7%)	2 (6.1%)	0.046
**Neuropsychological assessments**				
MMSE	26.94 ± 2.37	25.66 ± 2.24	28.64 ± 1.17	<0.001
**WHO-UCLA AVLT**				
Immediate recall	21.83 ± 6.92	17.52 ± 5.17	27.58 ± 4.27	<0.001
Delayed free recall	6.91 ± 3.37	4.75 ± 2.40	9.79 ± 1.07	<0.001
Recognition	10.00 ± 3.05	8.64 ± 2.96	11.82 ± 2.10	<0.001
BNT	22.62 ± 3.78	21.86 ± 4.07	23.64 ± 3.13	0.041
TMT B-A^*^	45.70 (27.00–114.35)	69.25 (33.07–150.00)	32.00 (20.00–62.50)	0.028
**MRI indices**				
FA				
Mean FA for global WM	0.41 ± 0.04	0.39 ± 0.04	0.43 ± 0.02	<0.001
ACR	0.35 ± 0.02	0.35 ± 0.02	0.34 ± 0.01	0.555
ATR	0.33 ± 0.03	0.32 ± 0.03	0.34 ± 0.02	0.001
CGC	0.41 ± 0.03	0.40 ± 0.03	0.42 ± 0.03	0.011
CGH	0.25 ± 0.03	0.25 ± 0.03	0.25 ± 0.02	0.915
CST	0.49 ± 0.03	0.49 ± 0.03	0.50 ± 0.02	0.026
FMA	0.48 ± 0.06	0.47 ± 0.07	0.50 ± 0.04	0.009
FMI	0.44 ± 0.05	0.43 ± 0.05	0.46 ± 0.04	0.008
IFO	0.40 ± 0.03	0.39 ± 0.03	0.41 ± 0.02	0.003
ILF	0.40 ± 0.03	0.39 ± 0.03	0.42 ± 0.02	0.003
PTR	0.40 ± 0.03	0.39 ± 0.03	0.41 ± 0.02	0.014
SLF	0.41 ± 0.04	0.40 ± 0.04	0.42 ± 0.02	<0.001
STR	0.41 ± 0.03	0.41 ± 0.03	0.41 ± 0.03	0.217
UNC	0.32 ± 0.02	0.32 ± 0.03	0.33 ± 0.02	0.003
Total GM volume, ml	574.05 ± 56.36	563.71 ± 56.58	587.82 ± 53.85	0.063
Total GM volume ratio^†^	0.3877 ± 0.0388	0.3753 ± 0.0414	0.4043 ± 0.0279	0.001
Mean hippocampal volume, ml	3.84 ± 0.21	3.76 ± 0.23	3.94 ± 0.12	<0.001
Mean hippocampal volume ratio^†^	0.0026 ± 0.0003	0.0025 ± 0.0003	0.0027 ± 0.0003	0.005
NbM volume, ml	1.69 ± 0.09	1.67 ± 0.09	1.72 ± 0.06	0.002
NbM volume ratio^†^	0.0011 ± 0.0001	0.0011 ± 0.0001	0.0012 ± 0.0001	0.013

*WMH, white matter hyperintensity; ApoE, apolipoprotein E; MMSE, Mini-mental State Examination; WHO-UCLA AVLT, WHO-UCLA Auditory Verbal Learning Test; BNT, Boston Naming Test; TMT B-A, Trail Making Test B-A; ATR, anterior thalamic radiation; ACR, acoustic radiation; CGC, cingulate gyrus part of cingulum; CGH, parahippocampal part of cingulum; CST, corticospinal tract; FMA, forceps major; FMI, forceps minor; IFO, inferior fronto-occi pital fasciculus; ILF, inferior longitudinal fasciculus; PTR, posterior thalamic radiation; SLF, superior longitudinal fasciculus; STR, superior thalamic radiation; UNC, uncinate*.

* *Values are medians (interquartile ranges). Mann–Whitney U tests were used for group comparisons*.

† *Corrected for ICV*.

### Predictors for Cognitive Performances

First, logistic regression was performed with the disease status (VCIND = 1 or NC = 0) as the dependent variable and z scores of mean FA for global WM and the volume ratios as the independent variables. Age, gender, education and ApoE ε4 status were included in the models as covariates. The results indicated that the mean FA value was the only independent predictor for cognitive decline (z score: OR = 4.649, 95%CI 1.576–13.712, *p* = 0.005). Furthermore, logistic regression was performed with z scores of FA for tract-specific WM and the volume ratios as the independent variables. In 13 tracts, only FA of CST (z score: OR = 2.081, 95%CI 1.078–4.016, *p* = 0.029) and UNC (z score: OR = 2.045, 95%CI 1.062–3.941, *p* = 0.032) were associated with the disease status. Then, to examine whether FA for global WM is a better predictor than tract-specific FA, we controlled the modified global FA that excludes the specific tract (CST or UNC) in the models (29). After controlling this global FA, the FA of CST (*z* score: OR = 1.984, 95%CI 0.932–4.221, *p* = 0.075) and UNC (z score: OR = 1.312, 95%CI 0.605–2.844, *p* = 0.492) could not predict the onset of cognitive impairment.

We then used the Spearman rank correlation to explore the correlations between the MRI predictors and cognitive performances. The results revealed that MMSE, immediate recall, delayed free recall and TMT B-A were all significantly correlated with FA value and brain volume. Recognition was only correlated with FA, and BNT scores were not correlated with any prediction parameter.

Based on these exploratory correlation analyses, we constructed multivariate linear regression models controlling for age, gender, education and ApoE ε4 status, with the scores of cognitive tests used as the dependent variables. For TMT B-A log transformations were used. The results are presented in Table 2. FA value and hippocampal volume were found to be independent predictors for MMSE. Immediate recall, delayed free recall, and recognition, however, were significantly associated with FA only. After controlling for possible confounding factors, TMT B-A scores were found to be not associated with FA or brain volume.

**Table 2:** The regression models for the neuropsychological assessments.

	**MMSE**			**Immediate recall**			**Delayed free recall**			Recognition^*^			**TMT B-A**		
	**β**	**95%CI**	***P***	**β**	**95%CI**	***P***	**β**	**95%CI**	***P***	**β**	**95%CI**	***P***	**β**	**95%CI**	***P***
z score of mean FA	0.452	0.349−1.792	0.004	0.464	1.109−5.310	0.003	0.600	1.076−2.973	0.000	0.386	0.385–1.970	0.004	−1.877	−0.265−0.008	0.065
z score of total GM ratio	−0.246	−1.398−0.232	0.158	−0.015	−2.473−2.271	0.933	−0.051	−1.243−0.900	0.751	-	-	-	−0.204	−0.235−0.068	0.274
z score of hippocampal ratio	1.076	0.182−4.917	0.035	0.159	−5.794−7.989	0.752	−0.233	−3.900−2.325	0.615	-	-	-	0.232	−0.368−0.561	0.679
z score of NbM ratio	−0.664	−3.902−0.758	0.183	0.130	−5.883−7.681	0.792	0.417	−1.658−4.469	0.363	-	-	-	−0.175	−0.527−0.383	0.752

* *Recognition was not correlated with brain volumes, thus brain volumes were not added in the regression model*.

### Associations Between FA and Brain Volumes

The linear regression models showed that FA was significantly associated with the corrected volumes of the total GM (β = 0.557, *p* < 0.001), the hippocampus (β = 0.444, *p* < 0.001) and the NbM (β = 0.394, *p* = 0.001). The comparison of brain volumes by tertiles of mean FA is shown in Table 3 and shows that those with lowest FA values also had the smallest volumes of total GM, hippocampus and NbM.

**Table 3:** Brain volumes of the study participants by tertiles of FA value.

	**Low FA**	**Middle FA**	**High FA**	***P***
Total GM volume, ml	557.39 ± 57.27	561.59 ± 44.31	604.14 ± 56.97	0.004
Total GM volume ratio	0.3555 ± 0.0392	0.3917 ± 0.0261	0.4156 ± 0.0242	<0.001
Mean hippocampal volume, ml	3.65 ± 0.22	3.87 ± 0.13	3.98 ± 0.09	<0.001
Mean hippocampal volume ratio	0.0023 ± 0.0002	0.0027 ± 0.0002	0.0028 ± 0.0002	<0.001
NbM volume, ml	1.63 ± 0.08	1.69 ± 0.08	1.75 ± 0.06	<0.001
NbM volume ratio	0.0010 ± 0.0001	0.0011 ± 0.0001	0.0012 ± 0.0001	<0.001

## Discussion

Although older adults with confluent WMHs have a relatively high risk of progression to VCI, the risk varies substantially among individuals. This study assessed the WM FA using DTI and brain volumes, including total GM, hippocampus, and NbM simultaneously. Our results indicated that the mean FA value for global WM was the only independent risk factor for the onset of VCI. The mean FA was significantly associated with both global cognitive function and memory. Additionally, a lower global mean FA was associated with smaller total volumes of GM, hippocampus and NbM. Compared to FA, brain volumes had a more limited predictive capability for cognitive impairment in the participants. Only the hippocampal volume was associated with global cognitive function, while the volumes of total GM and NbM were not associated with any cognitive performances.

FA detected by DTI is a common technique for evaluating the WM microstructure. FA decreases may be observed up to a decade prior to WMHs on routine MRI, and previous studies have demonstrated that patients with VCIND had decreased FA in all projection fibers, association fibers, and commissural fibers (30). In the present study, we calculated the mean FA value of 20 WM tracts to represent the global WM microstructure, as it has been suggested to be a better predictor for cognitive decline than tract-specific FA in older adults (29). Although in our study, we also calculated the tract-specific FAs, they were not associated with the risk of cognitive impairment after controlling the FA for global WM. Our study also suggested that when predicting the onset of global cognitive impairment, the mean FA for the whole-brain WM is better than tract-specific tracts. Our results showed that the mean FA value was significantly lower in the VCIND group compared with the NC group and it was the only substantial risk predictor for the onset of VCIND. Furthermore, the mean FA value was significantly associated with memory function. Recent studies have suggested that in clinically normal older adults a lower FA is associated with worse episodic and working memories (29, 31). However, both these prior studies and our own study did not find an association between FA and executive function, which is the primary impairment induced by VCI (32). Conversely, some other studies have reported correlations between FA and executive function (33, 34). A possible explanation for this discrepancy could be the influences of the confounding factors. In our study FA was initially correlated with executive function, however, after controlling for age, gender, education, and ApoE ε4 status no association was observed. The measurement methods for executive function may be another issue driving inconsistent results, as different scales were utilized in these various studies.

Several previous studies have tried to use MRI metrics, including the combination of measures for WMLs and GM volume, to predict cognitive decline. However, results have varied widely. Some studies show that the volumes of WMHs, the GM, and the hippocampus were all predictors for cognitive performances (5, 6), while others showed that the WMH volume was not associated with cognitive performances once atrophy measures were added into regression models (7). In contrast to these studies, our study used FA to evaluate WM integrity, which is a more sensitive evaluation than WMHs' volume. Furthermore, to exclude the diagnosis of mild cognitive impairment (MCI) of AD type, our inclusion criteria demanded no or very mild hippocampal or entorhinal cortex atrophy. Using these criteria, our study found that in multivariate models including both FA and brain volumes, FA was the strongest predictor for VCI and cognitive performances, and that brain volumes were not associated with cognitive function, except for the association between MMSE and the hippocampal volume. In addition to the association with the volumes of total GM and hippocampus, which have been investigated in previous studies, we also examined the predictive power of NbM volume, since the cholinergic system is important for cognitive function. However, our study did not find an association between NbM volume and cognitive performances. It has been suggested that the WM fiber projections of NbM may be a stronger contributor to cognitive function than NbM volume itself but the further evaluation will be needed (35).

Our study also showed that a lower global mean FA was associated with smaller total volumes of GM, hippocampus and NbM. One large-sample previous study enrolled 752 cognitively healthy participants and analyzed the explicit relationship between tract-specific WM and lobar GM volumes (26). The results suggested that tract-specific FA related to lobar GM volumes and for cognitive functions, memory was associated with lobar GM, while processing speed was related to tract integrity and lobar GM volumes. Compared to this previous study, our study aimed to identify the MRI predictors of VCI. According to this aim, the participants in our study had confluent WMHs and a relatively high risk for VCI. Then, disease status (VCIND or NC) and global cognitive function performance were the main analyzing measures in our study. Similarly, both of the studies controlled for non-brain factors and still identified the associations between MRI metrics and cognitive functions. This previous study controlled age, gender, education level, systolic blood pressure, and blood glucose level. In our study, age, gender, education, and ApoE gene ε4 status were controlled.

This present study has some limitations that need to note. First, this is a cross-sectional study. The causal relationships between WM microstructural integrity, GM volumes and VCI need to be verified in longitudinal studies. Furthermore, our sample size is relatively small. The small sample size could undermine the confidence of the results, and large sample studies are warranted. Another limitation is, as mentioned before, to exclude the confounding effect of MCI-AD, the hippocampal atrophy was limited as no or very mild. If the future studies use pathological biomarkers of AD, like β amyloid, to exclude the confounding diagnosis, the associations between WM microstructure and brain atrophy in people with confluent WMHs could be observed more precisely. Furthermore, with PANDA software, eddy-current induced distortion and head-motion are corrected by registering the DW images to the b0 image with an affine transformation. Although with this method, distortion still could not be completely removed. It was reported that distortion could lead to an erroneous increase in FA (36).

In conclusion, our study focuses on older adults with confluent WMHs on routine MRI and provides clarity to the possible predictive factors for cognitive decline. Our results suggest that the mean FA for global WM microstructural integrity is a superior predictor for VCI than the tract-specific FA and brain volumes. Furthermore, the impairment of WM microstructure was associated with worse memory and smaller volumes of the total GM, the hippocampus, and the NbM. Therefore, among patients who have confluent WMHs, we recommend DTI assessment as a valuable method for early identification of those at a higher risk of cognitive impairment.

## Data Availability Statement

The original contributions presented in the study are included in the article/supplementary material, further inquiries can be directed to the corresponding author/s.

## Ethics Statement

The studies involving human participants were reviewed and approved by Institutional Review Board of Xuanwu Hospital. The patients/participants provided their written informed consent to participate in this study.

## Author Contributions

YX, YT, and JJ designed this study. JY was responsible for data collection. AZ, FW, and CW enrolled participants and evaluated cognitive function. YX drafted the manuscript. All authors revised it and agreed the final version to be published.

## Conflict of Interest

The authors declare that the research was conducted in the absence of any commercial or financial relationships that could be construed as a potential conflict of interest.
